# Supplementary material for: Coherent Driving of a Single Nitrogen Vacancy Center by a Resonant Magnetic Tunnel Junction
Source: Nano Lett. 2024 Oct 30;24(45):14273–8. doi: 10.1021/acs.nanolett.4c03882 (PMC11565739; doi:10.1021/acs.nanolett.4c03882)
Supplement: Supplementary file 1 — nl4c03882_si_001.pdf [file nl4c03882_si_001.pdf]

Supporting Information

Coherent Driving of a Single Nitrogen Vacancy Center by a Resonant Magnetic Tunnel Junction

Gerald Q. Yan[1,2,+], Nathan McLaughlin[2,+], Tatsuya Yamamoto[3], Senlei Li[1], Takayuki Nozaki[3], Shinji Yuasa[3], Chunhui Rita Du[1,2,*], and Hailong Wang[1,*]

[1]School of Physics, Georgia Institute of Technology, Atlanta, Georgia 30332, USA
[2]Department of Physics, University of California, San Diego, La Jolla, California 92093, USA
[3]National Institute of Advanced Industrial Science and Technology (AIST), Research Center for Emerging Computing Technologies, Tsukuba, Ibaraki, 305-8568, Japan

[*]Corresponding authors: cdu71@gatech.edu; hwang3021@gatech.edu
[+]These authors contributed equally.

1. Relevant nitrogen vacancy spin physics

To achieve high field sensitivity and nanoscale spatial resolution, we employed diamond cantilevers containing single, optically addressable nitrogen vacancy (NV) centers for scanning NV magnetometry measurements.[1,2] Figure S1a displays a representative confocal image of a diamond cantilever hosting a single NV electron spin. The energy level of an NV center as a function of the external magnetic field B_{ext} applied along the NV spin orientation is presented in Fig. S1b. When the external magnetic field $B_{\text{ext}} > 0$, the initially degenerate $m_s = -1$ and $m_s = +1$ NV spin states experience a splitting by an energy gap of $2\gamma B_{\text{ext}}$, where γ represents the gyromagnetic ratio of NV centers. This "three-level" spin system can be optically addressed via the measured spin-dependent photoluminescence (PL). In comparison with the excited $m_s = 0$ state, the $m_s = \pm 1$ spin states are more likely to decay to the $m_s = 0$ ground state via a non-radiative intersystem crossing, emitting reduced PL. A typical NV electron spin resonance (ESR) spectrum is presented in Fig. S1c, where PL peaks corresponding to the two Zeeman split NV spin states are shown. The magnitude of the static magnetic field at the NV site B_{NV} parallel to the NV spin orientation can be obtained using the following equation:[3,4]

$$B_{\text{NV}} = \pi(f_+ - f_-)/\gamma \tag{1}$$

where f_+ and f_- correspond to the $m_s = +1$ and $m_s = -1$ NV ESR energies, respectively. The total field experienced at the NV site B_{NV} contains contributions from both the external magnetic field B_{ext} and the magnetic stray field B_s emanating from the magnetic tunnel junction (MTJ) device:

$$B_{\text{NV}} = B_{\text{ext}} + B_s \tag{2}$$

By subtracting B_{ext}, which can be obtained by additional NV measurements, from B_{NV}, the static stray field B_s produced by the MTJ device is quantitatively determined. Next, by scanning the diamond cantilever containing an NV center across the MTJ surface, the spatially varying B_s can be measured, as shown in Figure 2c of the main text.

2. Simulations of magnetic stray field pattern of an MTJ

Here, we present the detailed method used for micromagnetic simulations of static magnetic stray fields of the MTJ device presented in Fig. 2d of the main text. Generally, a static magnetization distribution $\mathbf{M}(\mathbf{R}')$ corresponding to a magnetic system determines its resultant stray field distribution $\mathbf{B}_s(\mathbf{R})$ as follows:[4,5]

$$\boldsymbol{B}_s(\boldsymbol{R}) = \int d^3\boldsymbol{R}'\mathcal{D}(\boldsymbol{R}, \boldsymbol{R}')\boldsymbol{M}(\boldsymbol{R}'), \tag{3}$$

where $\mathcal{D}(\boldsymbol{R}, \boldsymbol{R}') = -\nabla_{\boldsymbol{R}}\nabla_{\boldsymbol{R}'}(1/|\boldsymbol{R} - \boldsymbol{R}'|)$ is the magnetostatic Green's function tensor between coordinates $\mathbf{R} = (x, y, z)$ and $\mathbf{R}' = (x', y', z')$. Focusing on the magnetic field at the position of the NV center ($z = d_{\text{NV}}$), we perform Fourier transforms along the x and y directions, where translational symmetries are present, and $\mathbf{r} = (x, y)$ and $\mathbf{k} = (k_x, k_y)$:

$$\boldsymbol{B}_s(\boldsymbol{k}) = \int \boldsymbol{B}_s(\boldsymbol{r}, d)\, e^{i\boldsymbol{k}\cdot\boldsymbol{r}} d^2\boldsymbol{r},$$
$$\boldsymbol{M}(\boldsymbol{k}, z) = \int \boldsymbol{M}(\boldsymbol{r}, z)\, e^{i\boldsymbol{k}\cdot\boldsymbol{r}} d^2\boldsymbol{r}. \tag{4}$$

Similarly, we can obtain the Green's function tensor in the Fourier space for $\alpha, \beta = x, y$:

$$\begin{cases} D_{\alpha\beta}(\boldsymbol{k}, z') = -2\pi\big(k_\alpha k_\beta / k\big) e^{-k(d-z')}, \\ D_{\alpha z}(\boldsymbol{k}, z') = -2\pi i k_\alpha e^{-k(d-z')}, \\ D_{zz}(\boldsymbol{k}, z') = 2\pi k e^{-k(d-z')}. \end{cases} \tag{5}$$

where $k = |\boldsymbol{k}|$. This gives:

$$\boldsymbol{B}_s(\boldsymbol{k}) = \int D(\boldsymbol{k}, z')\boldsymbol{M}(\boldsymbol{k}, z')\, dz'. \tag{6}$$

Figure 2d of the main text shows the stray field pattern simulated using Eq. 6 with d_{NV} = 250 nm.

3. NV measurements of local oscillating magnetic stray fields

When the magnetic resonant frequency of the MTJ matches the NV ESR frequency, the magnetization dynamics in the MTJ generates oscillating magnetic fields which can drive transitions between two NV spin states, resulting in periodic oscillations of the spin state occupations, which are known as Rabi oscillations. The magnitude of the oscillating magnetic stray field transverse to NV-axis can be extracted from the NV Rabi oscillation frequency f_{Rabi} as follows:[6]

$$B_\perp = \frac{2\sqrt{2}\pi f_{\mathrm{Rabi}}}{\gamma} \tag{7}$$

where $\frac{\gamma}{2\pi}$ = 2.8 MHz/G is the electron spin gyromagnetic ratio. Figure S2a shows the measured Rabi oscillation frequencies at various points along the short axis (y-axis) of a resonant MTJ device with the vertical NV-to-sample distance d_{NV} = 250 nm. The input microwave power is 15 dBm for the presented measurements. Using Eq. 7, we can extract the spatial variation of $B_\perp$ as shown in Fig. S2b as well as Fig. 4d of the main text. The obtained experimental values agree with the theoretical calculations presented below.

4. Theoretical calculations of oscillating magnetic stray fields generated by voltage driven magnetic resonance of an MTJ

This section details the method used to calculate the amplitudes of oscillating magnetic stray fields produced by voltage controlled magnetic anisotropy (VCMA)-driven ferromagnetic resonance in an MTJ. Figure S3a depicts a schematic of the junction device wherein the magnetic reference ($Co_{56}Fe_{24}B_{20}$) and free ($Co_{40}Fe_{40}B_{20}$) layers are separated by an insulating tunneling barrier MgO. The lateral dimensions of the junction are 6 μm and 2 μm along the long and short axes, corresponding to the x and y axes, respectively. The thicknesses of the free and reference layers are 1 nm and 5 nm, respectively. In the analytical model, the free layer is assumed to occupy the volume Ω, defined as −3 μm ≤ x ≤ 3 μm, −1 μm ≤ y ≤ 1 μm, and −1 nm ≤ z ≤ 0, with its top surface lying in the x-y plane. An NV center is embedded within a diamond cantilever that is positioned on top of the junction device. The vertical distance between the NV and the free layer d_{NV} is ~250 nm. An external magnetic field B_{ext} is applied along the NV orientation defined as: $\mathbf{e}_{\mathbf{NV}}$ = [sin(54°), 0°, cos(54°)]. Throughout the calculations, we assume that the static magnetization of the reference layer lies parallel to the x-axis, while the static magnetization of the free layer aligns with the direction of the external magnetic field. The saturation magnetization, $4\pi M_s$, of the

free layer is approximately ~15 kG.[7] When a radiofrequency voltage is applied across the junction, the dynamic magnetization **M** of the free layer precesses around its equilibrium position, as illustrated by the blue dashed arrows in Figure S3a. The magnetic stray field $\mathbf{B}_s$ (**r**, *t*) at a location **r** generated by the time-dependent magnetization distribution, **M** (**r**', *t*), of the free layer at location **r**', can be expressed using the following equation:[8]

$$\boldsymbol{B}_s(\boldsymbol{r},t) = \int \left\{ \frac{3(\boldsymbol{r}-\boldsymbol{r}')[\boldsymbol{M}(\boldsymbol{r}',t)\cdot(\boldsymbol{r}-\boldsymbol{r}')]}{|\boldsymbol{r}-\boldsymbol{r}'|^5} - \frac{\boldsymbol{M}(\boldsymbol{r}',t)}{|\boldsymbol{r}-\boldsymbol{r}'|^3} \right\} d^3\boldsymbol{r}' \tag{8}$$

Here, we focus primarily on the transverse component, $B_\perp$ (relative to the NV axis), of the oscillating magnetic stray field along a linecut 250 nm above the free layer ($x = 0$ and $z = 250$ nm). $B_\perp$ is defined as $[B_{\max}(\mathbf{r}) - B_{\min}(\mathbf{r})]/2$, where $B_{\max}(\mathbf{r})$ and $B_{\min}(\mathbf{r})$ represent the maximum and minimum values, respectively, of the time-dependent magnetic stray fields perpendicular to the NV axis, $\mathbf{e}_{\mathbf{NV}} \times \mathbf{B}_s(\mathbf{r}, t)$. Figure S3b shows $B_\perp$ calculated along the linecut across the junction device illustrated by the black dashed lines ($x = 0$ and $z = 250$ nm) shown in Fig. S3a. At positions along the linecut for which $|y| \leq 1$ μm (corresponding to the dimension along the short axis of the junction device), $B_\perp$ exhibits a finite value as large as ~9 G. Outside this region, where $|y| > 1$ μm, the calculated magnetic stray field, $B_\perp$, exhibits a sharp decay and quickly reaches a vanishingly small value on a length scale of ~100 nm. Figure 4d in the main text shows the stray field pattern simulated using Eq. 8 with $d_{\mathrm{NV}} = 250$ nm.

References

(1) Li, S.; Huang, M.; Lu, H.; McLaughlin, N. J.; Xiao, Y.; Zhou, J.; Fullerton, E. E.; Chen, H.; Wang, H.; Du, C. R. Nanoscale Magnetic Domains in Polycrystalline Mn_3Sn Films Imaged by a Scanning Single-Spin Magnetometer. *Nano Lett.* **2023**, *23* (11), 5326–5333. https://doi.org/10.1021/acs.nanolett.3c01523.

(2) McLaughlin, N. J.; Li, S.; Brock, J. A.; Zhang, S.; Lu, H.; Huang, M.; Xiao, Y.; Zhou, J.; Tserkovnyak, Y.; Fullerton, E. E.; Wang, H.; Du, C. R. Local Control of a Single Nitrogen-Vacancy Center by Nanoscale Engineered Magnetic Domain Wall Motion. *ACS Nano* **2023**, *17* (24), 25689–25696. https://doi.org/10.1021/acsnano.3c10633.

(3) Rondin, L.; Tetienne, J.-P.; Hingant, T.; Roch, J.-F.; Maletinsky, P.; Jacques, V. Magnetometry with Nitrogen-Vacancy Defects in Diamond. *Rep. Prog. Phys.* **2014**, *77* (5), 056503. https://doi.org/10.1088/0034-4885/77/5/056503.

(4) Casola, F.; Van Der Sar, T.; Yacoby, A. Probing Condensed Matter Physics with Magnetometry Based on Nitrogen-Vacancy Centres in Diamond. *Nat. Rev. Mater.* **2018**, *3* (1), 17088. https://doi.org/10.1038/natrevmats.2017.88.

(5) Van Der Sar, T.; Casola, F.; Walsworth, R.; Yacoby, A. Nanometre-Scale Probing of Spin Waves Using Single Electron Spins. *Nat. Commun.* **2015**, *6* (1), 7886. https://doi.org/10.1038/ncomms8886.

(6) Hanson, R.; Dobrovitski, V. V.; Feiguin, A. E.; Gywat, O.; Awschalom, D. D. Coherent Dynamics of a Single Spin Interacting with an Adjustable Spin Bath. *Science* **2008**, *320* (5874), 352–355. https://doi.org/10.1126/science.1155400.

(7) Jen, S. U.; Chou, T. Y.; Lo, C. K. Impedance of Nanometer Thickness Ferromagnetic Co40Fe40B20 Films. *Nanoscale Res. Lett.* **2011**, *6* (1), 468. https://doi.org/10.1186/1556-276X-6-468.

(8) Zangwill, A. *Modern Electrodynamics*; Cambridge University Press: Cambridge, 2013.

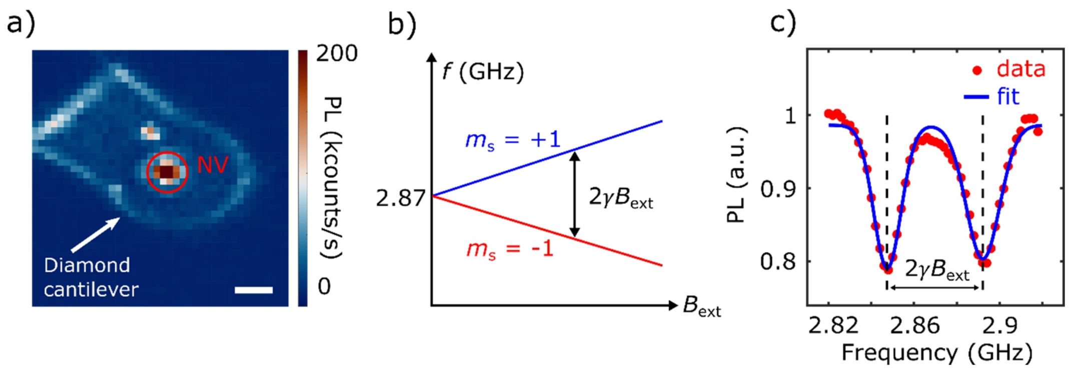

Figure S1. Single NV center-based ESR measurements. (a) A photoluminescence image of a patterned diamond cantilever containing a single NV center. The scale bar is 3 μm. (b) The energies of NV spin states as a function of an external magnetic field B_{ext} applied along the NV axis. (C) A representative optically detected magnetic resonance (ODMR) spectrum measured from an NV center contained in the diamond cantilever.

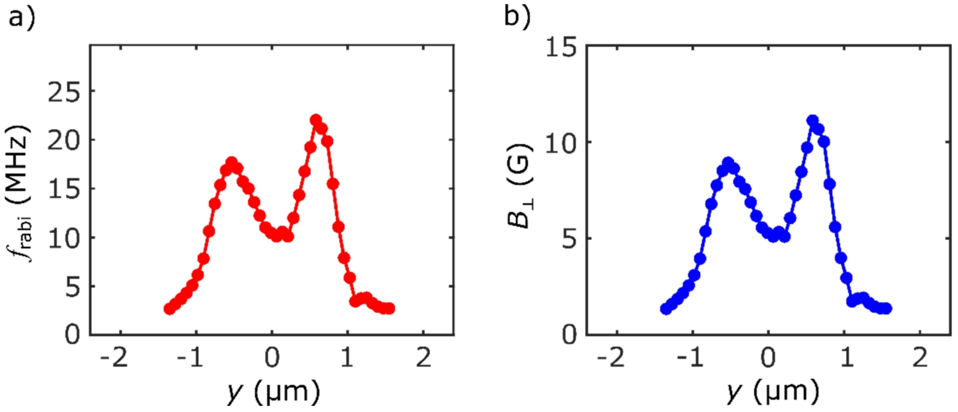

Figure S2. NV measurements of oscillating magnetic stray field. (a) The measured NV Rabi oscillation frequencies along a line of points across the short axis (y-axis) of an MTJ device. (b) Extracted spatial dependence (along the short axis) of the magnetic stray field, $B_\perp$, perpendicular to the NV spin orientation.

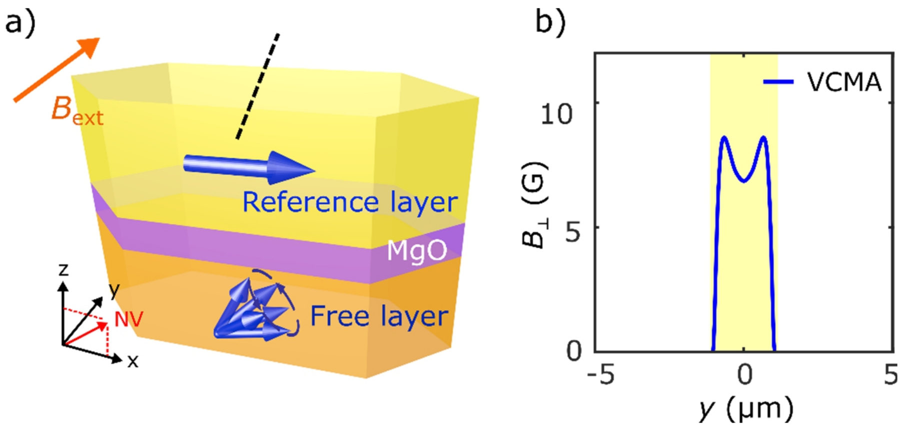

Figure S3. Calculation of magnetic stray field generated by a resonant MTJ. (a) Schematic of an MTJ device with reference, tunneling, and free layers. The black dashed line is 250 nm above the free layer (x = 0, z = 250 nm) and represents the linecut along which the spatially dependent magnetic stray field $B_\perp$ generated by the resonant free layer is calculated. The NV orientation is along [sin(54°), 0°, cos(54°)] as illustrated. (b) Calculated one-dimensional spatially dependent magnetic stray field $B_\perp$ (blue line) along the linecut (x = 0 and z = 250 nm). The yellow shaded area represents the lateral length scale of the magnetic pillar junction along y-axis.
